# Design and development of a new lentiviral based anti-HIV therapeutic vaccine

**DOI:** 10.1186/1742-4690-9-S2-P350

**Published:** 2012-09-13

**Authors:** M Rodriguez, E Sarry, A Bejanariu, L Casaban, S Abdalla, E Sabbah-Petrover, C Bauche

**Affiliations:** 1 Theravectys, Paris, France

## Background

Theravectys develops a new generation of prophylactic and therapeutic vaccines using optimized lentiviral vectors. It’s most advanced product, a therapeutic anti-HIV vaccinal treatment, will enter clinical Phase I/II within a few weeks. This vaccination will allow seropositive patients to gain an immunological status identical to the so-called “Functional Cured” patients who develop an efficient immunological response capable of controlling the infection without therapy.

## Methods

Vaccine candidates are integrative and self-inactivated live-recombinant lentiviral vectors. They encode an HIV antigen, under the regulation of a patented promoter that is preferentially induced in APC (generating of a strong, specific and long lasting T-cell immune response), and showing a basal level expression in all cells (allowing their elimination by the settled immune response).

Furthermore, Theravectys developed a vaccination regimen based on iterative immunizations with lentivectors encoding the same HIV transgene, relying on different VSV-G serotypes for pseudotyping without generating cross-neutralizing antibodies. These candidates were classified as “Live recombinant vectored vaccines” (EMA, 2011).

## Results

Theravectys set up an innovative manufacturing process combining high production yields, impurity profiles compatible with direct injections into humans and high immunogenicity.

Pilot and GMP batches have been manufactured and GLP preclinical studies (amongst which biodistribution, shedding and toxicity) performed, that showed the restricted diffusion of the vaccine candidates after injection and their fast disappearance within few weeks, correlated with an absence of macroscopic and microscopic toxicity.

## Conclusion

These data allowed the settlement of an anti-HIV therapeutic Phase I/II clinical trial that should receive the authorization of the French regulatory agency before the end of July.

This trial will be held in France and Belgium and plans the enrollment of 36 HIV-1 infected patients. Theravectys’ anti-HIV vaccine treatment will be assessed at three doses and safety, tolerability and immunogenicity compared to a placebo group. Results are expected by September 2014 with intermediary analyses in September 2013.

